# *Unmeasured* human transcription factor ChIP-seq data shape functional genomics and demand strategic prioritization

**DOI:** 10.1093/bfgp/elaf016

**Published:** 2025-09-30

**Authors:** Saeko Tahara, Haruka Ozaki

**Affiliations:** Bioinformatics Laboratory, Institute of Medicine, University of Tsukuba, Tsukuba 1-1-1 Tennodai, Tsukuba, Ibaraki 305-8577, Japan; Bioinformatics Laboratory, Institute of Medicine, University of Tsukuba, Tsukuba 1-1-1 Tennodai, Tsukuba, Ibaraki 305-8577, Japan; Center for Artificial Intelligence Research, University of Tsukuba, 1-1-1 Tennodai, Tsukuba, Ibaraki 305-8577, Japan; Laboratory for AI Biology, RIKEN Center for Biosystems Dynamics, 2-2-3 Minatojima-minamimachi, Chuo-ku, Kobe, Hyogo 650-0047, Japan

**Keywords:** TF ChIP-seq, transcription factor, regulatory TF prediction, unbiased large-scale data, unmeasured

## Abstract

Transcription factor (TF) chromatin immunoprecipitation followed by sequencing (ChIP-seq) is essential for identifying genome-wide TF-binding sites (TFBSs), and the collected datasets offer a variety of opportunities for downstream analyses such as inference of gene regulatory network and prediction for effects of single-nucleotide polymorphisms (SNPs) on TFBSs. Although TF ChIP-seq data continue to accumulate in public databases, comprehensive coverage of biologically relevant TF-sample pairs (i.e. combination of targeted TF and cell type) remains elusive. This is due to the need for TF-specific antibodies and large cell numbers, limiting feasible TF–cell type combinations. Moreover, ChIP-seq is measurable when the TF is expressed in the target cell type. Thus, defining the full space of biologically relevant TF–sample pairs—including both measured and unmeasured—is essential to assess and improve dataset comprehensiveness. Here, we investigated publicly available human TF ChIP-seq datasets and introduced the concept of *unmeasured* TF-sample pairs, defined as biologically relevant TF–sample combinations for which ChIP-seq experiments have not yet been performed. Notably, many expressed TFs in specific cell types remain unmeasured by ChIP-seq, affecting the coverage of regulatory regions revealed by TF ChIP-seq and genome-wide association study–SNP analyses. Furthermore, we propose practical strategies to efficiently supplement currently unmeasured data and discuss how these approaches can significantly enhance data-driven research. The database of unmeasured human TF–sample pairs is publicly accessible at https://moccs-db.shinyapps.io/Unmeasured_shiny_v1/, facilitating the systematic expansion of TF ChIP-seq datasets and thereby enhancing our comprehension of gene regulatory mechanisms.

## Introduction

Transcription factor (TF) chromatin immunoprecipitation followed by sequencing (ChIP-seq) is a technique that combines immunoprecipitation using antibodies specific to TFs with high-throughput sequencing technology to identify genome-wide TF-binding sites (TFBSs). International consortia, such as the Encyclopedia of DNA Elements (ENCODE) project [1], and carefully curated databases [2–5] have significantly contributed to the accumulation and accessibility of extensive ChIP-seq datasets. These collections of TF ChIP-seq data facilitate various applications, including enrichment analysis in promoter regions of differentially expressed genes (DEGs) or other genomic regions of interest [2–4, 6–8], reconstruction of gene regulatory networks [9–12], functional interpretation of disease-associated genomic regions [6, 13–16], assessment of single-nucleotide polymorphism (SNP) effects within TFBSs [17, 18], and integration into trans-omics analyses or artificial intelligence (AI)–driven drug discovery efforts for identifying pioneer TFs [19] and linking drugs to diseases [20].

Despite these valuable resources and their broad applications, there remains a critical yet often overlooked limitation: many biologically relevant TF–sample combinations remain entirely unmeasured by ChIP-seq, resulting in significant gaps in our understanding of the functional genomic landscape. Given that the human genome encodes over 1600 TFs [21], which are expressed across at least 400 distinct cell types [22], achieving full comprehensive coverage is inherently challenging. The incompleteness of current datasets primarily stems from two factors: (i) combinatorial complexity, as each ChIP-seq experiment targets a specific combination of TF and biological sample (i.e. cell type or tissue), creating immense number of potential TF–sample pairs; and (ii) technical and practical constraints, such as the limited availability of TF-specific antibodies and the necessity of ~1–10 million cells per experiment, which restrict individual laboratories to studying only a subset of TFs or cell types. Moreover, meaningful ChIP-seq experiments require the TF of interest to be expressed in the selected cell type. Thus, research efforts must strategically prioritize biologically relevant TF–sample pairs rather than attempting exhaustive coverage of all theoretical combinations. Consequently, when evaluating the comprehensiveness of TF ChIP-seq datasets, the exploration space should explicitly be defined as the collection of all biologically relevant TF–sample pairs, including both measured and unmeasured experiments.

A balanced and near-complete TF–cell-type atlas would replicate, for TF binding, the catalytic effect that community reference resources have had in genomics. Landmark consortia such as the Human Genome Project, ENCODE, and The Cancer Genome Atlas Program have shown that when data generation is decoupled from any single hypothesis and made openly available, the wider community repeatedly repurposes these data for unanticipated discoveries [1, 23, 24]. For ChIP-seq, systematic coverage would (i) enable reliable cross-TF comparisons, (ii) support the training of large-scale foundation models for regulatory genomics [25], and (iii) improve the annotation of noncoding disease variants in tissues that currently lack reference binding profiles. Because individual laboratories have little incentive to map low-visibility TFs, international consortia and publicly funded initiatives facilitate the execution of the missing experiments—an approach already proven viable by ENCODE and, more recently, by single-cell atlasing efforts. Advances in ChIP-seq protocols have steadily reduced costs to the point where systematic, high-throughput mapping is technically and financially feasible [26–30]. Such datasets will equip basic scientists to construct unbiased regulatory networks while giving clinicians and computational biologists stronger tools for variant interpretation and drug–target discovery. Nevertheless, despite substantial progress, the community still lacks a clear, quantitative picture of the present imbalance in TF coverage—a gap that must be resolved before a truly comprehensive atlas can be charted.

In this study, we introduce the concept of *unmeasured* TF–sample pairs, defined as biologically relevant combinations of TFs and samples for which ChIP-seq experiments have not yet been performed. We systematically investigate dataset biases arising from these missing data by quantifying the extent of unmeasured TF–sample pairs in the largest currently available human TF ChIP-seq database. We also evaluate their impact on downstream analyses, including the accuracy of upstream regulatory TF predictions and the interpretations of genome-wide association study (GWAS)–associated SNPs. Furthermore, we propose practical strategies to efficiently supplement currently unmeasured data and discuss how these approaches can substantially enhance data-driven genomic research.

## Results

### Past and current research attention strongly influences human transcription factor ChIP-seq data

**Figure 1:**
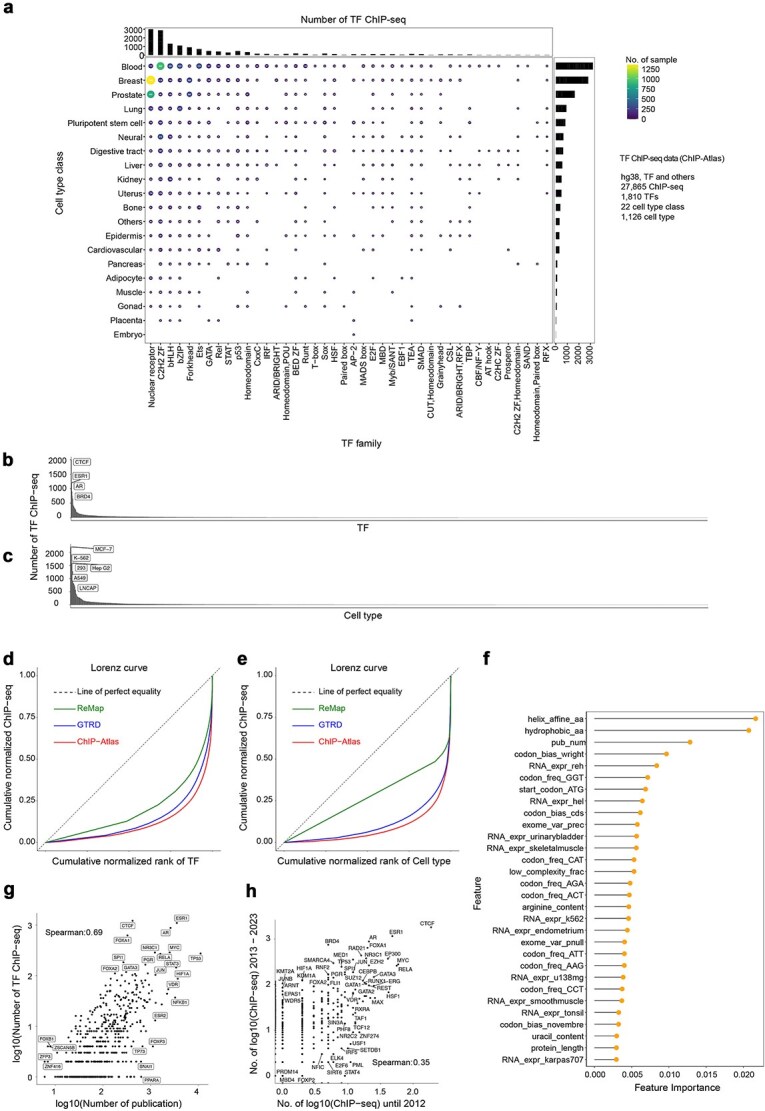
Past and current research attention strongly influences human TF ChIP-seq data. (a) Dot plot illustrating the number and proportion of human TF ChIP-seq experiments per TF family and cell type class. (b, c) Distribution of ChIP-seq experiments across individual TFs (b) and cell types (c). (d, e) Lorenz curves showing the cumulative distribution of ChIP-seq experiments across TFs (d) and cell types (e) in three databases: ChIP-Atlas, GTRD, and ReMap. The *x*-axis represents TFs or cell types ordered by the number of experiments, and the *y*-axis indicates the cumulative proportion of experiments. (f) Feature importance scores from the XGBoost model predicting the number of human TF ChIP-seq experiments based on 428 TF features (RNA and protein sequence features, publication counts; Supplementary Table 1). (g) Scatter plot demonstrating the positive correlation between the number of publications and ChIP-seq experiments per TF (Spearman correlation = 0.69). (h) Scatter plot demonstrating the positive correlation of ChIP-seq experiment counts per TF between earlier (before 2012) and later (after 2013) periods (Spearman correlation = 0.35).

To investigate the distribution of publicly available human TF ChIP-seq data across TFs and cell types, we utilized ChIP-Atlas [4], the ChIP-seq database containing the largest number of human ChIP-seq experiments. As of 4 October 2023, the human TF ChIP-seq dataset in ChIP-Atlas included 27 865 ChIP-seq experiments (SRX IDs; SRA Experiment accession) covering 22 cell type classes (1126 cell types) and 45 TF families (1810 target TFs or antigens). The number of ChIP-seq experiments and unique target TFs has steadily increased each year (Fig. S1a and b). However, the distribution of experiments was notably skewed toward specific TF families (e.g. C2H2 ZF, bZIP, bHLH), individual TFs (e.g. CTCF, ESR1, AR, BRD4), cell type classes (e.g. Blood), and specific cell types (e.g. MCF-7, K-562, HepG2) (Figs 1a–c and S1c and d). Among the cell type classes, Blood had the highest number of ChIP-seq experiments with 801 TFs, while Embryo had the fewest, with only 15 TFs. Further, within each cell type class, an uneven distribution of ChIP-seq experiments across individual cell types was observed (Fig. S1E and F).

To quantitatively assess this imbalance, we calculated the Gini coefficients for the number of ChIP-seq experiments targeting each TF and cell type (Methods). The Gini coefficient is a measure of inequality, ranging from 0 (perfect equality) to 1 (extreme inequality), and is commonly used to quantify distributional skew. The resulting Gini coefficients were 0.77 for TFs and 0.82 for cell types, indicating substantial inequality in experimental coverage (Fig. 1d and e). In contrast, when we assumed that all expressed TFs in each cell type had been measured by ChIP-seq (Methods), the Gini coefficient for TFs decreased to 0.47, highlighting the significant imbalance present in actual experimental datasets. Similar patterns of imbalance were observed in other ChIP-seq databases: Gene Transcription Regulation Database (GTRD) (TFs: 0.72, cell types: 0.77) [2] and ReMap (TFs: 0.60, cell types: 0.45) [3]. This suggests that the observed imbalance primarily reflects community-driven biases in experimental focus rather than the database-specific data collection biases. These findings underscore significant unevenness in the representation of target TFs and cell types within human ChIP-seq datasets.

To investigate the factors contributing to this observed imbalance, we developed a machine learning model (XGBoost [31]) to predict the number of TF ChIP-seq experiments based on 428 TF features, including features of RNA and protein sequence characteristics previously described by Stoeger *et al.* [32], and the number of publications associated with each TF (Methods, Fig. S1G, Supplementary Table 1). The model achieved high predictive accuracy, as evidenced by a Spearman correlation coefficient of 0.526 between predicted and observed experiment counts (Fig. S1h). Among all features, the number of publications was the third predictor (Fig. 1f) and demonstrating a strong positive correlation (Spearman correlation coefficient: 0.69) with ChIP-seq experiment counts (Fig. 1g). Given that publication frequency is a proxy for research attention [32], these results indicate that TFs receiving greater research attention are more frequently targeted by ChIP-seq experiments.

We further investigated whether the popularity of specific TFs in ChIP-seq experiments has shifted over time or whether certain TFs have remained consistently popular. Comparing the number of ChIP-seq experiments conducted before 2012 to those after 2013 for each TF revealed a positive Spearman correlation coefficient of 0.35 (Fig. 1h). This finding indicates a “rich-get-richer” effect, as reported for gene research [32, 33], where historically popular TFs continue to attract substantial research interest, maintaining their prominence in recent ChIP-seq studies.

### Potentially measurable ChIP-seq experiments remain unmeasured across various tissue types

**Figure 2:**
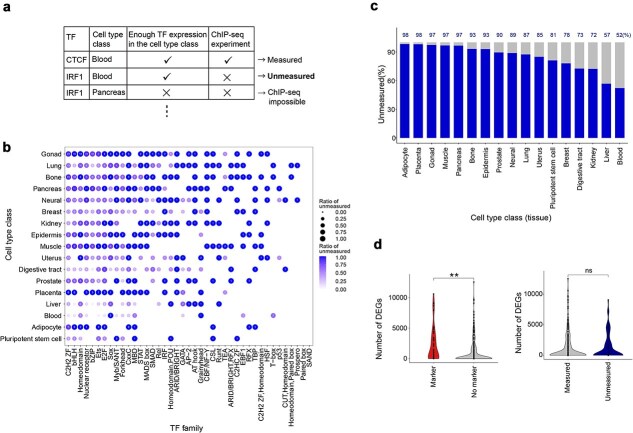
Potentially measurable ChIP-seq experiments remain unmeasured across various tissue types. (a) Schematic illustration defining *unmeasured* TF–sample pairs. (b) Dot plot showing the percentage of unmeasured but expressed TFs across TF families and cell type classes. The row is the cell type class, and the column is the TF family sorted by the number of unmeasured TFs. Each number inside the circle represents the number of unique unmeasured TFs in expressed TFs. Circle size and color indicate the proportion of unmeasured expressed TFs. (c) Proportion of unmeasured TFs among expressed TFs by cell type class. Numbers on the bar plot are the proportion of unmeasured TFs. (d) Comparison of DEG counts in TF knockout or knockdown experiments. Left: Cell-type-specific TF markers showed significantly higher DEG counts compared to nonspecific TFs (Wilcoxon rank sum test, *P* < .05). Right: No significant difference was found between measured and unmeasured TFs (Wilcoxon rank sum test, *P* = .86).

Although TF ChIP-seq data are unevenly distributed, the extent to which biologically relevant TF–sample combinations remain unmeasured has not been fully explored. To identify such missing yet potentially measurable TF–sample pairs in TF ChIP-seq coverage, we explicitly defined *unmeasured* TF-sample pairs (Fig. 2a). We defined expressed TF–sample pairs at the cell-type (cell-line) level. To capture cell-type-specific expression, we determined whether each TF was expressed by applying a step-function approach [34, 35] to its RNA-seq expression distribution compiled from 317 human cell lines to set a TF-specific expression threshold (Methods, Fig. S2a). This yielded a list of unmeasured TF–cell-type (cell-line) pairs (Fig. S2b). By aggregating across related cell types, we next defined expressed TF–cell type-class pairs and enumerated unmeasured TF–cell type-class pairs. The unmeasured pairs proved highly nonuniform across TF families and cell-type classes (Fig. 2b): for example, Blood and Liver classes contained fewer than 10 unmeasured TF-sample pairs per TF family, whereas Gonad and Bone classes exceeded 20 unmeasured pairs identified within the C2H2-ZF family—the family with the greatest overall ChIP-seq coverage. The fraction of unmeasured TFs remains substantial in every class, ranging from ~50% of expressed TFs in Blood to >80% in Adipocyte, Placenta, Gonad, Muscle, and Pancreas classes (Fig. 2c). These findings highlight substantial gaps, indicating that many biologically relevant ChIP-seq experiments remain unperformed.

Next, we examined whether these unmeasured TF–sample pairs might possess functional importance. To assess functional significance, we calculated the number of differentially expressed genes (DEGs) following the knockout (KO) or knockdown (KD) of TFs, comparing measured and unmeasured TF–sample pairs. First, to confirm that the number of DEGs serves as an appropriate metric for functional importance, we compared DEG counts between known cell-type-specific TF markers (TFs serving as markers in specific tissues or cell types) and nonspecific TFs. As expected, cell-type-specific TF markers exhibited significantly higher DEG counts than nonspecific TFs (Wilcoxon rank sum test, *P* < .05; Fig. 2d, left). Subsequently, when we directly compared the number of DEGs between measured and unmeasured TFs, no significant difference was observed (Wilcoxon rank sum test, *P* = .86; Fig. 2d, right). These results indicate that measured TFs are not necessarily associated with greater functional importance in terms of DEG count, suggesting that at least some unmeasured TF–sample pairs could indeed represent functionally important biological targets.

### Prioritization of candidate transcription factor–sample pairs for future ChIP-seq experiments

**Figure 3:**
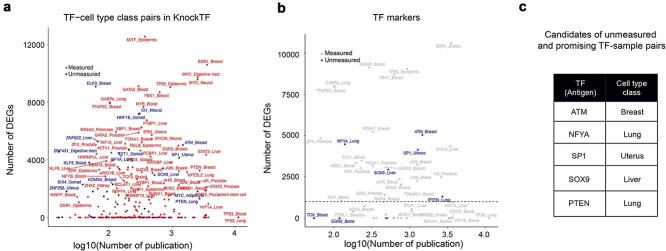
Prioritization of candidate TF–sample pairs for future ChIP-seq experiment. (a, b) Comparison of DEG counts from KO/KD experiments and associated publication numbers between measured and unmeasured TF–cell type class pairs (a) and specifically among TFMarker TFs (b). The dashed line indicates the threshold of 1000 DEGs. (c) Identified candidates (“*hidden gems”*)—highly biologically relevant but currently unmeasured TF–sample pairs recommended for prioritization in future ChIP-seq studies.

To systematically identify priority candidates for future ChIP-seq experiments among the *unmeasured* TF–sample pairs, we searched for “*hidden gems*”—unmeasured TF–sample combinations with high potential biological significance. First, we selected TF–sample pairs associated with over 1000 DEGs upon KO or KD of the TF [36], ensuring substantial biological impact (Fig. 3a). Next, we further narrowed down these candidates by identifying TFs recognized as known TFMarkers (cell-type- or tissue-specific TFs used as markers), thus emphasizing both biological relevance and clinical importance [37] (Fig. 3b). Through this filtering, we pinpointed five notable unmeasured TF–sample pairs exhibiting >1000 DEGs upon TF knockout: ATM-Breast, NFYA-Lung, SP1-Uterus, SOX9-Liver, and PTEN-Lung (Fig. 3c).

For instance, the ATM gene is well-known as the third most frequently mutated gene in hereditary breast and ovarian cancer (HBOC) after BRCA1 and BRCA2. ATM deficiency has been shown to lead to abnormal transcriptional responses to estrogen, including activation of the oncogene c-MYC [38]. Despite ATM’s established significance in breast cancer pathology, no ATM ChIP-seq experiments have been conducted specifically in breast tissue, although such studies have been performed in other tissues. This example validates the importance of our identified “*hidden gems*,” suggesting that prioritizing these TF–sample pairs for future ChIP-seq experiments would substantially enhance our understanding of crucial regulatory networks in disease pathogenesis and cellular differentiation.

### Diversity of targeted transcription factors influences downstream analyses using transcription factor ChIP-seq datasets

The extensive accumulation of ChIP-seq data provides foundational resources for predicting regulatory TFs [39–42] and assessing the functional impact of GWAS-associated SNPs in TFBSs [17, 18]. Our previous results highlighted significant imbalances and gaps in current human TF ChIP-seq datasets, particularly regarding coverage across specific TFs and cell type classes. Such imbalances raise concerns about their potential impact on the accuracy of downstream analyses that rely on these datasets. Therefore, we investigated how uneven representation of TF ChIP-seq data might affect large-scale analyses.

**Figure 4:**
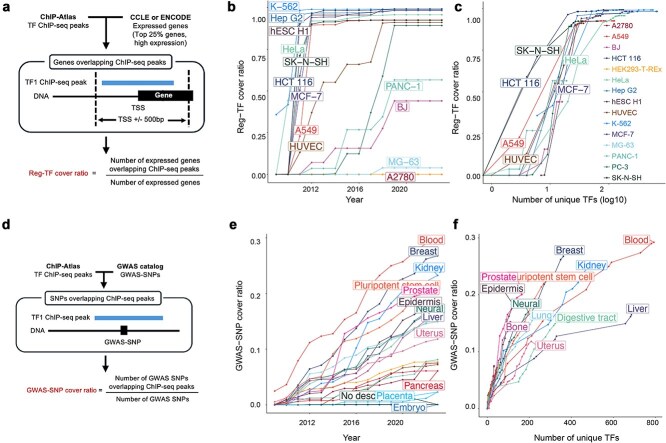
Diversity of targeted TFs influences downstream analyses using TF ChIP-seq datasets. (a) Overview of the calculation method for the regulatory TF (Reg-TF) cover ratio. (b) Annual changes in Reg-TF cover ratios across different cell lines (each color/line represents a distinct cell line). (c) Relationship between the number of measured unique TFs and the Reg-TF cover ratio. Each dot represents one cell line per year. (d) Overview of the calculation method for the GWAS-SNP cover ratio. (e) Annual changes in GWAS-SNP cover ratios across different cell lines (each color/line represents a distinct cell line). (f) Relationship between the number of measured unique TFs and the GWAS-SNP cover ratio. Each dot represents one cell line per year.

First, we assessed how comprehensively existing human TF ChIP-seq datasets cover regulatory TFs across various cell lines. For each cell line, we calculated the proportion of expressed genes having at least one TF ChIP-seq peak near their transcription start sites (TSS ± 500 bp), defined as the “regulatory TF (Reg-TF) cover ratio” (Methods, Fig. 4a). A higher Reg-TF cover ratio implies greater availability of data for accurately identifying regulatory TFs. Analysis of annual trends in Reg-TF cover ratios revealed substantial differences among cell lines. For example, K-562, a widely used leukemia cell line, consistently showed one of the highest Reg-TF cover ratios, indicating that most expressed genes in K-562 are supported by TF ChIP-seq data near their TSSs (Fig. 4b). In contrast, PANC-1, a pancreatic cancer cell line with fewer ChIP-seq experiments, had a considerably lower Reg-TF cover ratio—approximately 40% of expressed genes lacked nearby TF ChIP-seq peaks. This suggests that accurately predicting regulatory TFs for expressed genes in PANC-1 remains challenging, given the currently available data. Furthermore, we found a clear positive relationship between the number of measured unique TFs in a cell line and its Reg-TF cover ratio (Fig. 4c). Thus, the diversity of measured TFs by ChIP-seq significantly influences the reliability of regulatory TF predictions within each cell line.

Next, we evaluated the extent to which GWAS-identified SNPs are represented within the current TF ChIP-seq dataset. For each cell line, we calculated the “GWAS-SNP cover ratio,” defined as the proportion of GWAS-identified SNPs overlapping at least one TF ChIP-seq peak measured in the corresponding cell or tissue type (Methods, Fig. 4d). Analysis of annual trends in the GWAS-SNP cover ratio showed notable differences across tissues. For example, the Blood class, which has extensive ChIP-seq coverage (Fig. 1a), reached a GWAS-SNP cover ratio approaching 0.3 by 2023. Conversely, the Pancreas class, with fewer ChIP-seq experiments, remained below a GWAS-SNP cover ratio of 0.1 (Fig. 4e). Additionally, a positive correlation was observed between the number of measured unique TFs and the GWAS-SNP cover ratio for each cell line (Fig. 4f). These findings underscore the importance of considering TF diversity in ChIP-seq datasets when analyzing SNP impact, emphasizing that broader TF coverage is critical for more accurate and comprehensive interpretations.

### Feasibility of alternative strategies for conducting transcription factor ChIP-seq experiments

Our analysis of Reg-TF and GWAS-SNP cover ratios indicates that diversity in targeted TFs critically enhances the quality of downstream analyses utilizing TF ChIP-seq data. This observation raises an important question: What measurement strategy would maximize these cover ratios efficiently, particularly during the initial phases of data collection?

**Figure 5:**
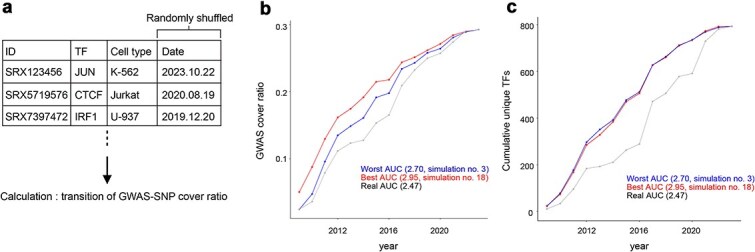
Feasibility of alternative strategies for conducting TF ChIP-seq experiments. (a) Schematic overview of the simulation examining optimal TF ChIP-seq measurement sequences to maximize the GWAS-SNP cover ratio. (b, c) Annual trends of GWAS-SNP cover ratios (b) and cumulative number of unique TFs (c) under simulated random measurement orders. Higher AUC indicates better early performance. Red lines represent the simulation order with the highest AUC, blue lines represent the lowest AUC, and gray lines depict the actual historical measurement order.

To explore this, we performed simulations to identify an optimal ordering of TF ChIP-seq experiments that would most rapidly increase the GWAS-SNP cover ratio in the early stages (Fig. 5a). Specifically, we simulated alternative measurement sequences by randomly rearranging the actual chronological order of ChIP-seq experiments conducted in the Blood cell type class, evaluating performance through the area under the curve (AUC) metric of the annual GWAS-SNP cover ratio trend. A higher AUC represents a quicker improvement in coverage during earlier years.

Our simulations identified several alternative experimental orders yielding higher AUC values compared to the original measurement sequence (Figs 5b and S3a). Sub-sampling analysis confirmed that these randomized orders yielded significantly higher AUCs than the real acquisition order (Welch’s *t*-test, *P* < .01) (Fig. S3b and c, Methods). Importantly, these high-performing alternative sequences consistently included a greater diversity (higher cumulative number) of unique targeted TFs measured in early stages (Figs 5c and S3d). These findings suggest that prioritizing ChIP-seq experiments targeting a diverse array of TFs early on is essential to rapidly and efficiently enhance analytical quality. Even when the total number of ChIP-seq experiments is constrained, this diversified measurement strategy significantly improves the effectiveness of TF ChIP-seq datasets in downstream analyses.

## Discussion

In this study, we systematically examined the comprehensiveness and biases within current human TF ChIP-seq datasets, revealing substantial imbalances in the coverage across TFs and cell types (Fig. 1). These biases primarily stem from uneven research attention toward specific genes, often driven by skewed publication frequencies of extensively studied “superstar genes” such as TP53 and EGFR [43, 44] (Fig. 1f and g). Similar biases have been widely recognized in other biological datasets, including drug–target interactions [45] and protein–protein interactions [46], where they are known to impact the validity and accuracy of computational analyses, machine learning predictions, and downstream interpretations [47, 48].

Importantly, we identified numerous biologically relevant but currently *unmeasured* TF–sample pairs (Figs 2 and 3). This underscores a significant limitation of existing ChIP-seq datasets: even highly expressed and likely functional TFs are not uniformly measured across relevant cell types. Our findings demonstrate that these coverage gaps substantially influence the reliability of downstream analyses, including regulatory TF predictions and interpretations of disease-associated genetic variations identified by GWAS (Fig. 4). Furthermore, simulation results emphasized that prioritizing measurement of diverse TFs in early stages of data collection stabilizes analysis outcomes and mitigates biases introduced by uneven datasets (Fig. 5).

While many meta-scientific studies [32, 49–52] have analyzed research biases toward specific genes or topics using scientific literature and patent databases, comprehensive assessments of such biases within large-scale omics datasets, including ChIP-seq, remain relatively limited. Our work addresses this gap by specifically analyzing biases and missing data within human TF ChIP-seq datasets.

We note several limitations of our study and outline future directions. First, the observed coverage imbalance may partly reflect “survivor bias”—TF–cell-type pairs lacking high-affinity antibodies or that are otherwise intractable seldom yield usable ChIP-seq data and therefore never enter public repositories, so pursuing such unmeasured pairs without accounting for these hidden failures could waste resources. Second, our definition of TF expression relies on the expression thresholds applied to RNA-seq distributions across 317 cell lines annotated in both the Human Protein Atlas and ChIP-Atlas; this approach may potentially overlook low-level or context-specific TF activity and exclude additional cell types present only in ChIP-Atlas, reducing resolution and potentially obscuring subtle lineage- or state-specific patterns. Future work will integrate more comprehensive ontology-based mapping and single-cell expression resources to enable higher-resolution analyses that capture additional unmeasured functional TF–cell-type relationships. Third, we used the number of DEGs as a proxy for functional importance, yet DEG counts are sensitive to KD methods (e.g., small Interfering RNA [siRNA] versus Auxin-Inducible Degron system [AID]), recovery time, and cell context and are not linearly proportional to regulatory impact. A more realistic prioritization scheme should combine DEG information with orthogonal evidence—including motif conservation, TF-expression levels, chromatin-accessibility footprints, and proximity to disease-associated variants—to generate a composite score that accounts for both biological relevance and experimental constraints. Finally, ChIP-seq peak density itself can be biased by GC content, open-chromatin artifacts, and antibody quality, so high apparent promoter coverage does not necessarily indicate functional binding. Addressing these limitations will be essential for translating our *in silico* findings into efficient, biologically meaningful experimental plans.

Computational methods predicting TFBSs from gene expression, ATAC-seq data, and TF motifs have been proposed as alternatives to ChIP-seq experiments. While these computational methods achieve relatively high accuracy (auPR; Area Under the Precision-Recall Curve ~0.8) for TFs with stable motifs (e.g. CTCF), their performance significantly declines (auPR ~0.2) for TFs with less stable motifs [39, 40]. Thus, conducting actual TF ChIP-seq experiments remains essential, particularly given the growing dependence of large-scale models on high-quality training datasets [39]. Recent technological advancements, such as CRISPR Epitope Tagging ChIP-seq (CETCh-seq) [26] and multiplexed ChIP-seq methods like ChIP-DIP [27], now offer practical solutions for previous limitations related to antibody specificity, availability, and experimental scalability. Additionally, robotic automation has significantly streamlined ChIP-seq library preparation, increasing experimental throughput and feasibility [28–30].

Nevertheless, despite these technological advancements, conducting experiments for all potential TF–sample pairs remains impractical. Strategic prioritization of future experiments is therefore crucial. To facilitate informed prioritization, we provide an online resource containing a comprehensive list of unmeasured TF–sample pairs (https://moccs-db.shinyapps.io/Unmeasured_shiny_v1/). Integrating advanced experimental methods and computational prioritization strategies can accelerate balanced data acquisition and comprehensive exploration of transcriptional regulatory networks.

In conclusion, our study presents a systematic framework to identify and address biases and missing data in human TF ChIP-seq datasets, providing actionable strategies to improve dataset comprehensiveness. Enhancing the diversity and coverage of TF ChIP-seq data will significantly advance our understanding of gene regulatory mechanisms and strengthen the robustness of biomedical research dependent on these essential resources.

## Materials and Methods

### Data preparation

#### ChIP-seq peak data

The peak calling data of human TF ChIP-seq experiments (hg38) were obtained from the ChIP-Atlas database (https://chip-atlas.org/).

#### ChIP-seq metadata

The metadata of human TF ChIP-seq data in ChIP-Atlas [4, 53] (https://chip-atlas.dbcls.jp/data/metadata/experimentList.tab, experimentList.tab) was downloaded on 4 October 2023. The metadata in GTRD [2] (http://gtrd20–06.biouml.org/downloads/20.06/metadata/, ChIP-seq.metadata.txt) and ReMap 2022 [3] (https://remap.univ-amu.fr/download_page, remap2022_all_macs2_hg38_v1_0.bed) were also downloaded. The annotations of DNA-binding domains and TF families were obtained from CIS-BP [54].

### Publication data

We downloaded the gene2pubmed data from the File Transfer Protocol server (ftp://ftp.ncbi.nlm.nih.gov/gene/DATA/gene2pubmed.gz) and filtered human data by the National Center for Biotechnology Information (NCBI) Taxonomy ID (9606).

### Gene feature data

The data of 427 *z*-scored gene features over 12 948 genes were obtained from Table S2 of Stoeger *et al.* [32]. We added the log10-transformed and *z*-scored publication number from gene2pubmed to the data, resulting in the 428 *z*-scored gene feature matrix. We used 902 TFs for model training, as gene feature data (all 428 features) were available only for these TFs.

### Transcription start site data

The TSS annotations of human genes were downloaded from the UCSC table browser [55] (genome: human, assembly: GRCh38/hg38, group: Genes and Gene Predictions, track: NCBI RegSeq, table: UCSC RefSeq (RefGene)). RefSeq IDs were converted to gene symbols using the R package bioMart [56] (2.38.0). The TSS annotations were extended to +/− 500 bp using bedtools [57] (v2.30.0).

### Genome-wide association study-single-nucleotide polymorphism data

GWAS summary statistics were downloaded from the GWAS catalog [58] (All associations v1.0), comprising 270 440 unique rs numbers (GWAS SNPs).

### RNA-seq data

RNA expression data for 1206 human cell lines were obtained from the Human Protein Atlas [59] (https://www.proteinatlas.org/about/download#single_cell_type). We downloaded “rna_celline.tsv.zip.”

### Knock transcription factor, transcription factor marker data

DEGs in TF-knockout/knockdown experiments were obtained from KnockTFv2.0 [36](https://bio.liclab.net/KnockTFv2/index.php). We manually downloaded 616 human DEG lists of 351 TFs. TF marker data were obtained from “All_TFmarkers.txt” from the TF-Marker database [37] (https://bio.liclab.net/TF-Marker/).

### Data analysis

#### Integration of cell type class and cell type category among different databases

Annotations of cell type class and cell type in ChIP-Atlas were manually integrated with annotations of tissue name in other databases (RefEx, KnockTF, TF marker, and Human Protein Atlas) (Supplementary Table 2).

### Definition of expressed transcription factors

We standardized and matched cell line names between the Human Protein Atlas (1206 cell lines) and ChIP-Atlas (1098 cell types) datasets using Jaro–Winkler similarity with additional digit consistency checks with an in-house R script, resulting in the 317 common cell lines. Expressed TF–cell-type pairs were identified by fitting a step function to each TF’s RNA-seq expression distribution across 317 cell lines and classifying the expression as “expressed” or “not expressed” with the StepMiner algorithm [34, 35] (Fig. S2a). These expressed TF–cell-type pairs were then aggregated into expressed TF–cell type class pairs: a TF was labeled “expressed “in a given cell type class when at least one of its constituent cell types met the expression threshold.

### Calculation of the Gini index as a measure of imbalance in transcription factor ChIP-seq data

The Gini index for the number of ChIP-seq across TFs and cell types was calculated by using `ineq` R package (version 0.2.13). Expressed TFs in each cell type were defined as in Definition of Expressed Transcription Factors.

### Machine learning

We removed duplicates and unavailable features from 433 gene features and used the remaining *z*-scored 428 gene features for 902 TFs for the prediction of log10-transformed number of ChIP-seq experiments. The predictive model was built using XGBoost (version 2.0.0) in Python (version 3.8.17). The dataset was split into a training set (90%) and a test set (10%) 400 times. For each spilit, the model’s performance was evaluated on the test set using root mean squared error (RMSE). Finally, the median RMSE across the 400 randomizations was reported for each TF.

### Calculation of regulatory transcription factor cover ratio

Expressed genes were defined as the top 25% highly expressed genes per cell line. TSS regions were defined as TSS +/− 500 bps. For the TSS region of each expressed gene, “bedtools intersect” was used to determine whether the TSS region overlapped with any TF ChIP-seq peaks from ChIP-Atlas. The “regulatory-TF (Reg-TF) cover ratio” was calculated per cell line and year and defined as follows:

*Reg-TF cover ratio = Number of expressed genes with any ChIP–seq peaks measured until target year/Number of expressed genes*

### Calculation of genome-wide association study-single-nucleotide polymorphism cover ratio

For each GWAS SNP, “bedtools intersect” was used to determine whether the GWAS-SNP overlapped with any TF ChIP-seq peaks from ChIP-Atlas. The “GWAS-SNP cover ratio” was calculated per cell type class and one year and defined as follows:

GWAS-SNP cover ratio = Number of GWAS-SNPs with any ChIP-seq peaks measured until target year/Number of GWAS-SNPs.

### Simulation of genome-wide association study-single-nucleotide polymorphism cover ratio

ChIP-seq data conducted in Blood were used for the simulation of the GWAS-SNP cover ratio. ChIP-seq IDs with deposited dates were prepared, and the dates were randomly shuffled, calculating the GWAS-SNP cover ratio until the target year. The simulation was repeated 100 times. For statistical analysis, sub-sampling experiments (90% and 50%) for each of the randomized acquisition order simulations (best performance top3; simulation no. 42, 11, 18, and worst performance top3; simulation no. 1, 3, 37) were conducted, and AUCs were computed for each sub-sample. Welch’s *t*-test was applied to compare the distributions with that of the real acquisition order.

### Statistical analysis of the number of differentially expressed genes in transcription factor-knock-out experiments

The number of DEGs in TF-KO or KD experiments between TF marker and non-TF marker or between measured TF and unmeasured TF was compared by the Wilcoxon rank sum test.

### Filtering unmeasured but potentially functional ChIP-seq

Measured combinations of TFs and cell type class in KnockTF were filtered, and then, combinations with >1000 DEGs in TF-knockout and TF marker in TF marker database were selected as candidates for unmeasured but potentially functional ChIP-seq.

### Ethics approval and consent to participate.

Not applicable.

### Consent for publication

All authors have approved the manuscript for submission.

### Declaration of generative AI and AI-assisted technologies in the writing process

During manuscript preparation, the authors used ChatGPT (GPT-4o, o3, and GTP-4.5) (OpenAI, CA, USA), a large language model, for English translation, grammar correction, and stylistic refinement. The AI tool was applied to individual sentences and paragraphs but was not used to generate new content or modify scientific conclusions. All content was thoroughly reviewed and edited by the authors, who take full responsibility for the manuscript’s accuracy, originality, and integrity.

**Key Points**

- We systematically investigated comprehensiveness and biases in current human TF ChIP-seq datasets, emphasizing the importance of identifying missing but measurable TF ChIP-seq experiments.
- We introduced the concept of *unmeasured* TF-sample pairs, defined as biologically relevant combinations of TFs and samples for which ChIP-seq experiments have not yet been performed.
- We demonstrated that increasing the diversity of TF ChIP-seq data significantly enhances our understanding of gene regulatory mechanisms and strengthens biomedical research relying on these datasets.

## Supplementary Material

Supplementary_rev1_elaf016

Supplementary_table1_elaf016

Supplementary_table2_elaf016

Supplementary_table3_Fig2B_unmeasured_elaf016

## Data Availability

The codes and data are available on the GitHub repository. (https://github.com/bioinfo-tsukuba/Unmeasured).

## Notes

**Saeko Tahara** received an MD from the University of Tsukuba in 2024. She is a resident at the University of Tsukuba Hospital and investigates bioinformatics-driven biomedical research at the university’s Institute of Medicine.

**Haruka Ozaki** received a Ph.D. from University of Tokyo in 2015. He is Associate Professor at the Institute of Medicine, University of Tsukuba. His interests include bioinformatics and research automation.
