# Supplementary material for: Unmeasured human transcription factor ChIP-seq data shape functional genomics and demand strategic prioritization
Source: Brief Funct Genomics. 2025 Sep 30;24:elaf016. doi: 10.1093/bfgp/elaf016 (PMC12479113; doi:10.1093/bfgp/elaf016)
Supplement: Supplementary_rev1_elaf016 [file supplementary_rev1_elaf016.pdf]

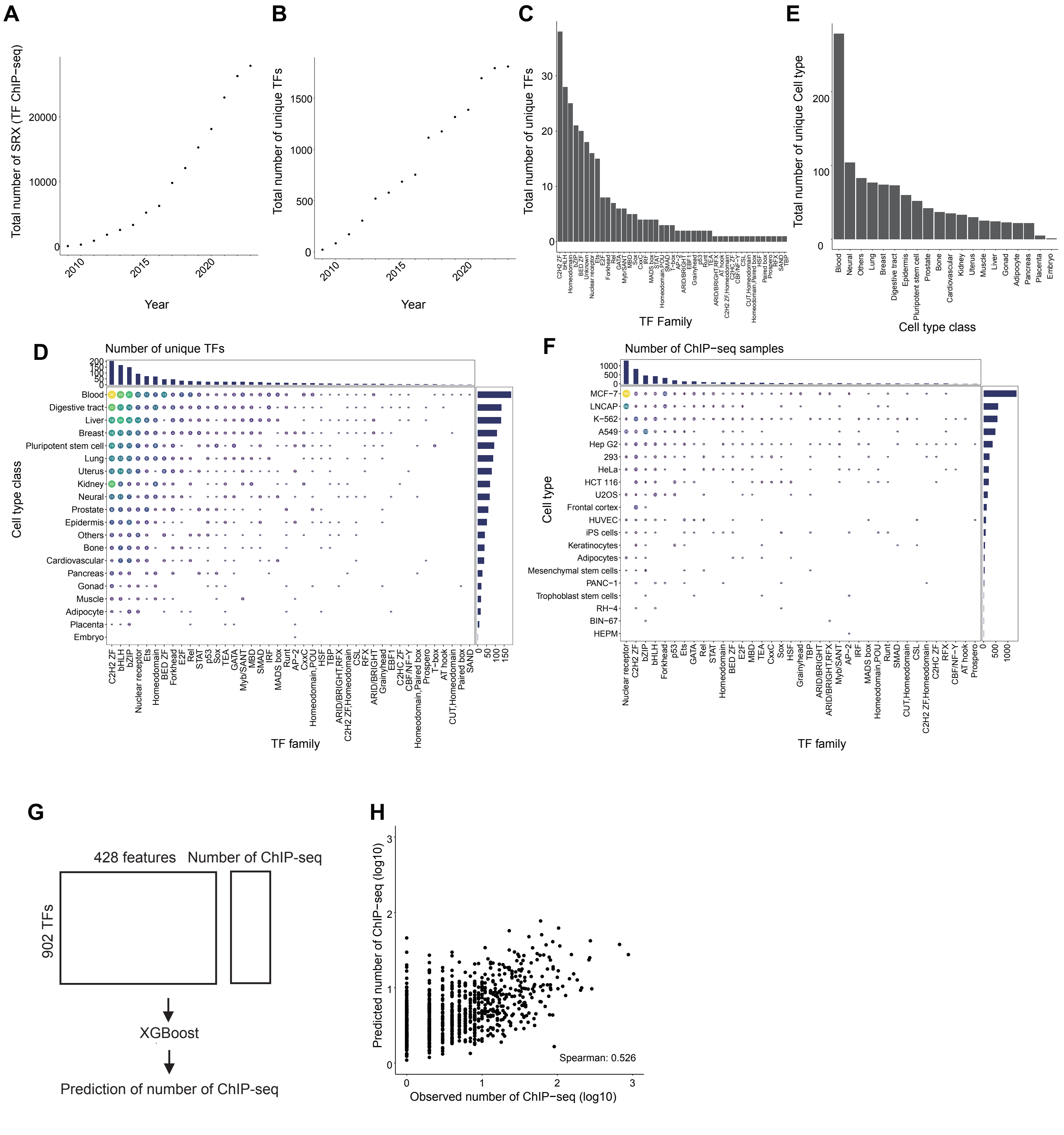

Figure S1 Global landscape and predictive modeling of human TF ChIP-seq coverage across time, TF families, and cell type classes.

A and B: Annual transition of the number of ChIP-seq experiments and unique targeted TFs. **C:** Number of unique TFs in each TF family. **D:** Dot plot of the number and ratio of unique target TFs across TF family and cell type class. **E:** Number of unique cell types in each cell type class. **F:** Dot plot of number and ratio of human TF ChIP-seq experiments across TF family and cell type. Representative cell types with maximum number of ChIP-seq experiments in each cell type class were selected. **G:** Illustration of modeling approach and prediction of number of human TF ChIP-seq experiments for each TF by 428 gene features. **H:** Scatter plot between observed number of ChIP-seq and predicted number of ChIP-seq by machine learning. Each point indicates each TF.

Tahara et. al., **Supplementary Figure 1**

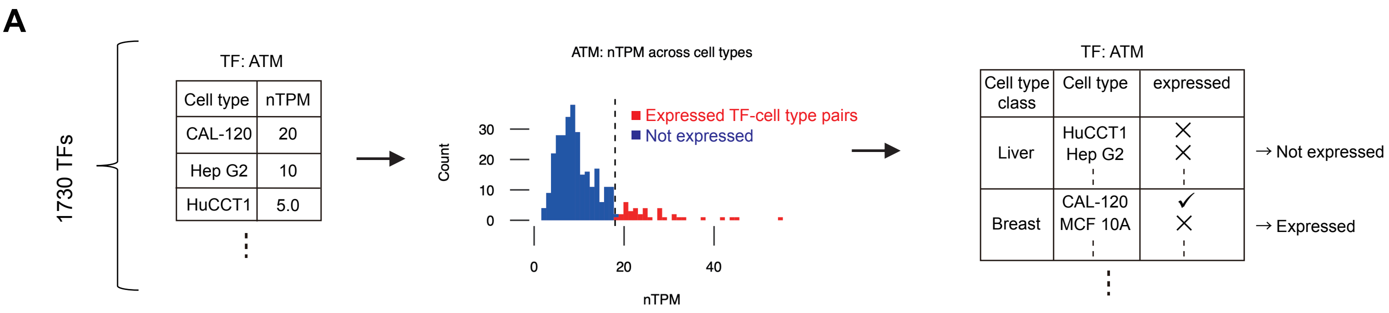

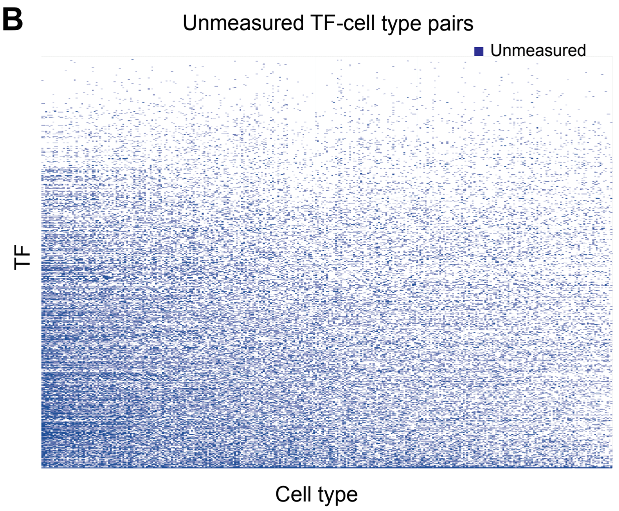

Figure S2 Definition of expressed TF and unmeasured TF-cell type pairs.

A: Schematic illustration defining expressed TF-cell type / TF-cell type class pairs. **B**: Heatmap of unmeasured TF-cell type pairs. Each row is TF and each column is cell type. Blue dot indicates unmeasured TF-cell type pair.

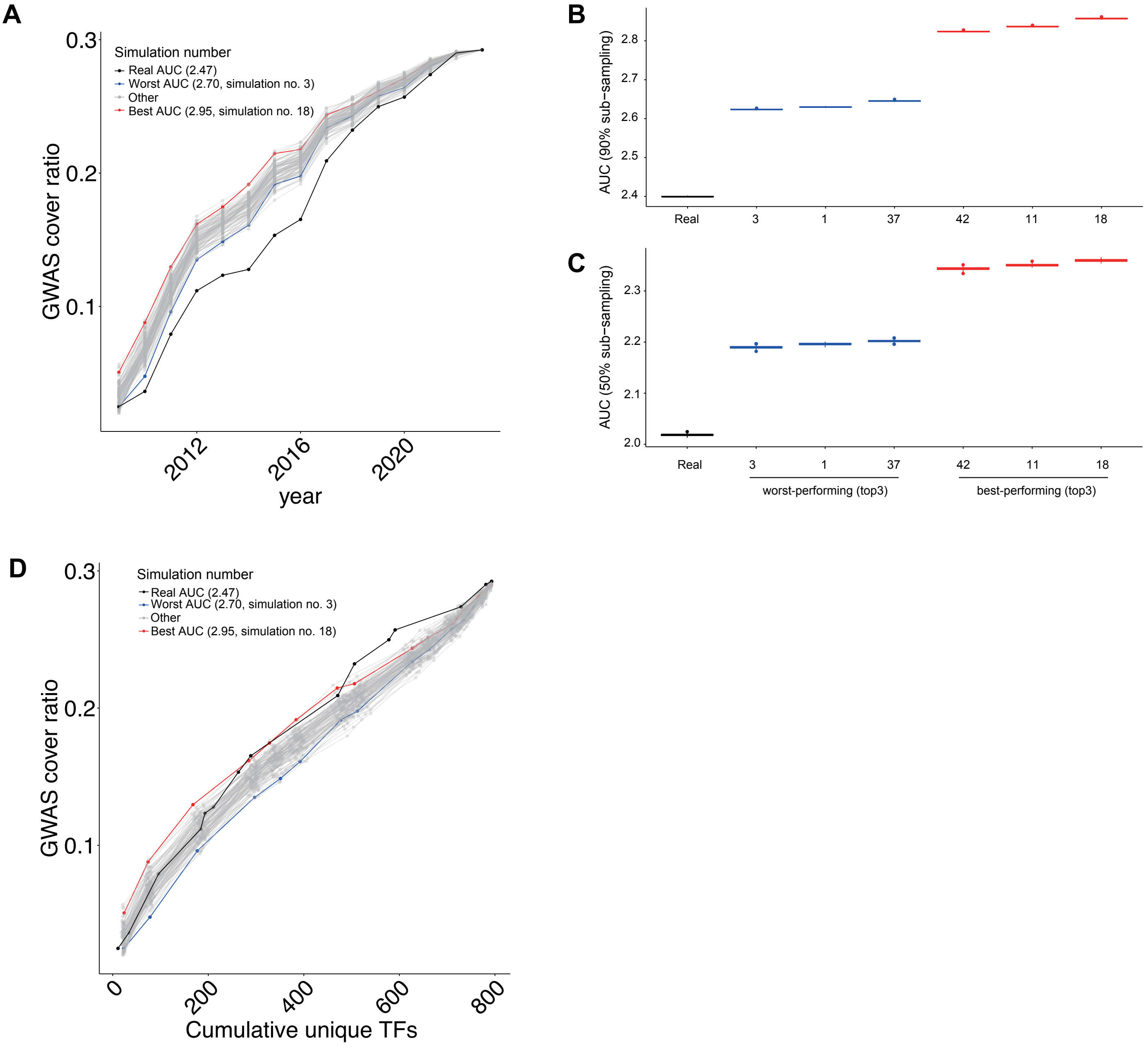

Figure S3 Simulation of GWAS-SNP coverage shows the impact of ChIP-seq acquisition order on cumulative performance.

A and D: Simulation of GWAS SNP cover ratio when the order of ChIP-seq experiments is randomly shuffled 100 times. GWAS-SNP cover ratio is shown against year (**A**) and the cumulative number of unique TFs (**D**). A high AUC indicates the high performance of the GWAS-SNP cover ratio. The red line is the highest AUC score, the blue one is the lowest AUC, the black one is the real transition, and the gray one is the other simulation. **B and C:** AUCs were calculated from 90% (**B**) and 50% (**C**) sub-samples of the real acquisition order and six representative randomized simulations: the three best-performing (red; simulation No. 42, 11, 18) and three worst-performing (blue; No. 1, 3, 37) simulation orders). In both conditions, all randomized simulations yielded significantly higher AUCs than the real acquisition order (Welch's t-test, $p < 0.01$)
